# Intimate partner violence amongst Brazilian lesbian women: mental health indicators, social support and minority stress

**DOI:** 10.1186/s41155-025-00371-0

**Published:** 2025-12-05

**Authors:** Julia de Oliveira Chotgues, Thaís de Castro Jury Arnoud, Luísa Fernanda Habigzang

**Affiliations:** Department of Psychology, Pontifical Catholic University of Rio Grande do Sul (PUCRS), Ipiranga Avenue, 6681, Partenon, Porto Alegre, 90619900 Brazil

**Keywords:** Intimate partner violence, Sexual and gender minorities, Lesbian, Mental health, Social support

## Abstract

**Background:**

The intimate partner violence (IPV) among lesbian women is an invisible problem in Brazil due to discrimination and social stigma. Most national studies focus on understanding violence in relationships between women and men, leaving a gap in research addressing this type of violence and its consequences for lesbian women. This study aims to identify the main types of IPV experienced by Brazilian lesbian women, compare mental health indicators, perceptions of social support, and minority stress levels between lesbian women with and without a history of IPV, and examine predictors of victimization in intimate relationships.

**Methods:**

Descriptive analyses, t-tests, and logistic regression analyses were performed. A total of 550 lesbian women participated, with a mean age of 25.31 years old (SD = 5.83).

**Results:**

Women with a history of IPV have worse mental health outcomes and lower perceptions of social support networks. In addition, concealment of sexuality, experiences of stigma, age, and social support were identified as predictors of IPV.

**Conclusion:**

The findings of this study highlight how IPV can further jeopardize the mental health of Brazilian lesbian women, exacerbating symptoms of stress, depression, and anxiety. Discriminatory and stigmatizing acts amplify the effects of IPV, as violence is perpetrated on both social and individual levels.

## Introduction

Violence in intimate relationships is one of the main forms of violence against women, representing a global public health issue (WHO, 2021). This type of violence can cause short-, medium-, and long-term harm, affecting women’s physical, mental, sexual, and reproductive health (WHO, 2021). Most research on intimate partner violence (IPV), both nationally and internationally, focuses on relationships between men and women. As a result, studies on IPV in LGBTQ + relationships (lesbian, gay, bisexual, transgender, queer persons) are scarce (e.g., Gehring & Vaske, 2017; Harden et al., 2022; Longobardi & Ribeira, 2017; Miller & Irvin, 2016; Santoniccolo et al., 2021; Whitfield et al., 2021), and even fewer focus specifically on relationships among lesbian women (e.g., Furukawa et al., 2022; Lewis et al., 2018; Santos et al., 2019). This gap may be due to the stigma and prejudice faced by non-heterosexual people in society, which also affects scientific research and production (Miller & Irvin, 2016; Santos, 2012; Santos et al., 2019). Moreover, considering the dynamics between the Global North and Global South in knowledge production, the erasure becomes even more pronounced: there is a notable gap in the literature on lesbian Latin American women and their experiences of violence in general. Therefore, research is urgently needed to explore the typologies, dynamics, and mental health effects of IPV in this population.

Studies on intimate partner violence (IPV) in relationships between women indicate that it can manifest as physical, psychological, sexual, and other forms of violence, often occurring simultaneously (Osorio et al., 2020; Whitfield et al., 2021). In Brazil, current legislation on domestic violence defines five types of violence against women: (a) Physical violence: any act that harms physical integrity or health; (b) Psychological violence: conduct that causes emotional harm, lowers self-esteem, impairs development, or humiliates; (c) Sexual violence: any act that forces a woman to witness, engage in, or maintain unwanted sexual activity through intimidation, threats, or use of force; preventing the use of contraceptive methods; or coercion into marriage, pregnancy, abortion, or prostitution; (d) Patrimonial violence: retention, subtraction, or partial or total destruction of personal belongings, work tools, documents, assets, valuables, or financial resources; (e) Moral violence: acts that constitute slander, defamation, or insult (Brazilian Federal Law nº 11.340, 2006).

The typologies and dynamics of IPV, however, are usually derived from studies focused on relationships between women and men. The contributions of intersectionality — a critical social theory widely developed by Black feminists (e.g., Collins and Crenshaw) — are particularly useful in understanding the dynamics of IPV within lesbian communities. Since IPV disproportionately affects socially marginalized individuals and is inherently rooted in unequal power relations, it is essential to consider intersecting characteristics such as race, income, age, gender, and sexual orientation (Harden et al., 2022; Whitfield et al., 2021) to fully comprehend how this phenomenon impacts lesbian couples. For instance, black lesbian women experience compounded stressors related to gender, race, and sexual orientation (Harden et al., 2022). In Brazil, epidemiological data show that intimate partner violence most frequently affects Black women (52.1%), with physical violence being the most common (84%) (Secretaria de Vigilância em Saúde, 2020). Age is another key factor: younger women are more likely to suffer abuse than older women (WHO, 2021), indicating that age can shape power dynamics in relationships between women (DiStefano, 2009; Hassouneh & Glass, 2008).

Lesbian women belong to the group of sexual and gender minorities. Meyer (2003) describes minorities as individuals who occupy a position of non-compliance with the social structure and norms established by the dominant class. Lesbian women, like other non-heterosexual women, challenge the heteronormative culture, which dictates that intimate relationships should exclusively involve cisgender women and cisgender men, invalidating other possibilities and formats of intimate connections. The Minority Stress Theory (MST) was first developed by Brooks (1981) to understand the impacts of violence experienced by black lesbian women due to stigma and prejudice stemming from gender violence, racism, and homophobia. Meyer (1995) expanded on this model, applying it to gay men, and later generalized it to encompass all individuals impacted by the dominant culture, including ethnic, racial, sexual, gender, and socioeconomic minorities (Meyer, 2003).

The Minority Stress (MS) stipulates that the impacts of prejudice and stigma processes can arise from distal and proximal contexts. In the distal context, stressors are external and independent of the individual, stemming from the social environment in which they are embedded. Within this context lies the concept of imposed stigma (1), defined as any form of violence experienced due to sexual orientation, such as physical aggression. In contrast, the proximal context involves stressors that depend on individual characteristics. These include internalized homonegativity (2), characterized by aversive feelings toward one’s own sexual orientation, and the concealment of sexual orientation (3), which occurs when an individual hides or omits their sexual identity (Meyer, 1995, 2003). These three elements represent specific stressors faced by sexual and gender minorities, which are compounded by general stressors experienced by the broader population, such as unemployment or low-income status (Meyer, 2003). Consequently, lesbian women experience unique stress processes, which must be considered to better understand the dynamics of IPV within these relationships.

In Brazil, some studies have examined the effects of MS on the LGBTQ + population. Paveltchuk et al. (2020) investigated the impacts of MS on levels of subjective well-being (SWB) and mental health among lesbian and bisexual women. Their findings indicated that experiences of stigma significantly affect both mental health and SWB, with internalized homonegativity impacting SWB and the concealment of sexual orientation affecting mental health outcomes. In another study, Paveltchuk and Borsa (2019) analyzed the effects of MS among lesbian, gay, and bisexual (LGB) individuals. Contrary to their 2020 findings, this research found that internalized homonegativity did not significantly influence mental health outcomes in their sample. These conflicting results highlight the need for further studies to deepen the understanding of how the MS model applies to Brazilian and broader Latin American contexts.

Nevertheless, lesbian women who experience IPV also face societal oppression, which worsens mental health and well-being outcomes (Santos, 2012). Scientific evidence indicates that it is not sexual orientation itself but rather the chronic discrimination and stigmatization faced by non-heterosexual individuals that contribute to these negative effects. Specifically, lesbian women possess an identity that is delegitimized by heteronormative cultural norms, and their relationships are often unrecognized, resulting in dual oppression based on both gender and sexual orientation (Santos, 2012; Souza et al., 2021a, b). In Brazil, the lesbian community continues to endure intense stigmatization and social prejudice. A recent study analyzing violence against lesbians in Brazil reported a 50% increase in cases of interpersonal violence between 2015 and 2022 (Firmino & Matias, 2024). Black lesbian women accounted for 56% of these reports, likely due to the compounded stressors of racism, sexism, and homophobia. Consequently, they are at greater risk of developing mental health issues, particularly in the context of violence and trauma (VPI), including depression, anxiety, substance abuse, emotional dysregulation, and other mental health disorders (Miller & Irvin, 2016; Lewis et al., 2018; Trombetta & Rollé, 2024).

Under this perspective, a specific stressor of IPV experiences in same-sex relationships is the threat of revealing the partner’s sexual orientation. This dynamic is used by the aggressor as a tool to exert power, resulting in significant psychological distress (DiStefano, 2009). Even if the scientific literature indicates that higher levels of outness is associated with well-being between youth LGBQ people (Shilo et al., 2015), disclosing one’s sexual orientation without their permission can lead to suffering and stress for the individual, and threats about this can mean a forced revelation. Lesbian women who have their sexual orientation hidden may choose not to reveal their experience of IPV for fear of being revictimized by those who could provide support (Furukawa et al., 2022). This can favor the development of a double closet: hiding one’s sexuality and hiding their violent relationships (Santos, 2012; Topa, 2010).

The support of significant individuals, such as friends and family, plays a crucial role in breaking the cycle of IPV and serves as a protective factor for mental health (Furukawa et al., 2022; Zamora et al., 2021). Beyond helping survivors cope with violence, the literature highlights that a perceived positive support network reduces the likelihood of developing depressive symptoms in LGB individuals (Miller & Irvin, 2016). Additionally, a sense of belonging to the LGBTQ + community can buffer the impacts of stigmatization (Meyer, 2003) and mitigate the consequences of IPV. In cases where family members are unaccepting, support networks comprising friends and LGBTQ + community members often take on the role of a “family of choice” (Shilo et al., 2015). However, research by Furukawa and colleagues (2022) found that individuals with higher levels of internalized homonegativity were less likely to seek help from their support networks, regardless of the severity of violence experienced. This reluctance may stem from fear of disclosure, revictimization, or the concealment of sexual orientation, reinforcing the concept of the double closet (Santos, 2012; Topa, 2010). Moreover, the same study revealed that lesbian women with a lower perception of social support had higher rates of IPV. Interestingly, socioeconomic status did not emerge as a determinant of perceived social support or the prevalence of IPV, suggesting that violence can occur across all social strata (Furukawa et al., 2022).

It is also essential to address support from a formal perspective, particularly the services that constitute the formal support network for women with a history of IPV, such as protection and assistance services. Although Brazilian legislation guarantees the right to protection for women experiencing domestic, family, and intimate relationship violence, irrespective of sexual orientation (Brazilian Federal Law nº 11.340, 2006), lesbian women often face barriers in accessing this support due to their sexual orientation. One of the primary obstacles is institutional prejudice, which delegitimizes lesbian relationships and hinders these women from receiving adequate assistance from the formal support network (Luz & Gonçalves, 2015; Santos et al., 2019). As a result, women in these situations may be hesitant to seek help from such services, fearing discrimination or the inability of the system to resolve their cases effectively (Santoniccolo et al., 2023).

Given that most of the evidence cited above comes from studies with samples from the Global North, and considering the significant gap in the literature regarding the Brazilian context, this study aims to identify the main types of IPV among lesbian women, compare mental health indicators, perceptions of social support, and stress levels between lesbian women with and without a history of IPV, and investigate whether sociodemographic characteristics (age, race, and social class), social stigma, internalized homonegativity, concealment of sexuality, and perception of social support are predictors of victimization in intimate relationships. Based on the literature reviewed, the hypotheses guiding this research are as follows: (1) The most prevalent form of violence among lesbian couples will be psychological violence; (2) Lesbian women with a history of IPV will report lower levels of perceived social and mental health support and higher levels of minority stress compared to those without a history of IPV; (3) Sociodemographic characteristics (age, race, and social class), indicators of minority stress, and perceptions of social support will predict experiences of victimization in intimate relationships.

## Method

### Design

Quantitative, transversal.

### Participants

This study included 550 cisgender women who identified as lesbians. Ages ranged from 18 to 58 years, with a mean age of 25.31 years (SD = 5.83). Participants identified themselves as white (*n* = 390), brown (*n* = 105), black (*n* = 48), yellow/Asian (*n* = 1), Indigenous (*n* = 2), and other (*n* = 4) - categories based on the Brazilian Institute of Geography and Statistics (IBGE - Instituto Brasileiro de Geografia e Estatística). Most participants resided in the South (*n* = 234) and Southeast (*n* = 173), followed by the Northeast (*n* = 98), Center-West (*n* = 31), and North (*n* = 14). The majority were single (*n* = 414) or married (*n* = 89), with the sample also including divorced (*n* = 9), separated (*n* = 2), and other marital statuses (*n* = 36). Additionally, most participants reported being in a current relationship (*n* = 365) and not having children (*n* = 528). Socioeconomic income ranged from up to one minimum wage (*n* = 66), one to two minimum wages (*n* = 141), two to four minimum wages (*n* = 160), four to six minimum wages (*n* = 77), to over six minimum wages (*n* = 106). Most participants were undergoing mental health care (*n* = 311), such as psychotherapy and/or psychiatric treatment.

To be included in the study, participants had to be 18 years old or older, identify as lesbian, and have experienced at least one intimate relationship with another woman. Thus, those who did not meet the research criteria were excluded from the study.

### Instruments

(A) Sociodemographic data questionnaire: this instrument seeks to investigate information regarding age, sexual orientation, race/ethnicity, education, marital status, employment status, presence of children, residence, monthly income, use of alcohol or other substances, religious belief, amongst others.
(B) Intimate Partner Violence Questionnaire: The questionnaire was developed based on typologies described in the Brazilian law regarding domestic violence, assessing moral, psychological, patrimonial, physical and sexual aggressions within the sample (Brazilian Federal Law nº 11.340, 2006). At first, there was a general question to investigate whether the participant considers herself to have already experienced some form of violence in an intimate relationship to which the participant could answer *yes* or *no*. Then, subtypes of IPV were presented, each one followed by examples. The participants could indicate which forms of violence they suffered after reading the examples, also answering *yes* or *no*.
(C) Depression, Anxiety and Stress Scale (DASS-21): instrument originally created by Lovibond and Lovibond (1995), translated into European Portuguese by Apóstolo, Mendes, and Azeredo (2006), and later adapted and validated for the Brazilian context by Vignola and Tucci (2014). The DASS-21 aims to assess depression, anxiety, and stress symptoms. It consists of 21 items and three subscales of 7 items, which aims to identify symptoms experienced by the individual in the previous week through a Likert scale ranging from 0 (“did not apply at all”) to 4 (“applied very much, or most of the time”). In the adaptation and validation for Brazil, good internal consistency was found for each subscale (Cronbach’s alpha of 0.92 for depression, 0.90 for stress, and 0.86 for anxiety).
(D) Multidimensional Scale of Perceived Social Support (MSPSS): instrument developed by Zimet et al. (1988) and translated, adapted, and validated for the Brazilian context by Martins et al. (2017). It aims to assess three main dimensions of perceived support, which are: family, friends, and other significant people. It consists of 12 items, divided into three dimensions, and answered using a Likert scale, being 1 (“I strongly disagree”) and 7 (“I strongly agree”). The Cronbach’s alpha indicates high precision in the results, being 0.93 for friends, 0.91 for family, and 0.90 for other significant people.
(E) Lesbian, Gay and Bisexual Minority Stress Assessment Protocol (PEM-LGB-BR) – Female Version (Lesbian and Bisexual Women): Costa, Paveltchuk, Lawrenz, Vilanova, Borsa, Damásio, Habigzang, Nardi, and Dunn (2020) conducted the cross-cultural adaptation and validation for the Brazilian context. The protocol consists of three measures: internalized homonegativity scale (7 items), sexuality disclosure scale (4 items), and stigma experience scale (7 items). The protocol has two versions: one for lesbian and bisexual women and another for gay and bisexual men. It has good internal consistency rates, with a Cronbach’s alpha of 0.72. The subscales also showed internal adequacy, with 0.79 for internalized homonegativity, 0.76 for sexuality disclosure, and 0.67 for stigma experiences.

### Data collection procedures and ethical considerations

Data collection was online, through the Qualtrics platform. An online disclosure card was made for social networks, such as Instagram and Facebook, containing the necessary information about participation requirements, the presentation of the research, and its purpose. On Instagram, pages from specific communities of lesbian women and the LGBTQ + public were contacted and asked for support in publicizing the study, in order to increase the reach of the research to the desired public. The collection took place from March to May 2022.

This study was approved by the Research Ethics Committee of the university responsible for the research group. As a form of ethical care for participants who could be in a situation of violence, information about the women’s protection network was presented at the end of the questionnaire, so that they could seek support. In addition, the contacts of the responsible researchers were provided to assist in any necessary referrals.

### Data analysis

The data analysis was performed using the Statistical Package for Social Science (SPSS) software, version 28.0.1.1(15). Descriptive analyses were performed to verify means, standard deviation, percentages, and frequencies to characterize the sample and to identify the most frequent types of IPV amongst lesbian women. In order to meet the objective of comparing the levels of perceived social support, mental health indicators, and stress levels of minorities in lesbian women with and without a history of IPV, Student’s t-tests were performed for independent samples. We assessed data normality using the Kolmogorov-Smirnov and Shapiro-Wilk tests. The variance homogeneity assumption was evaluated using Levene’s test. Bootstrapping procedures (1000 resampling; 95% *CI BCa*) were performed to obtain greater reliability of the results, to correct deviations from normality in the sample distribution and differences between the sizes of the groups, and also to present a confidence interval of 95% for differences between means (Haukoos & Lewis, 2005).

Finally, we performed a logistic regression analysis using the enter method. The analysis aimed to explain how socio demographic variables, perception of social support, substance use, history of domestic violence in the family, mental health monitoring, and elements of minority stress could be related to the experience of suffering IPV (suffered or did not suffer). We performed correlation analyses, and those that showed significant associations with IPV were included in the model.

## Results

Regarding the history of IPV, 216 participants (39.3%) reported previous experience. However, after being presented with examples of situations related to the five types of violence (psychological, physical, sexual, patrimonial, and moral), 324 participants (58.9%) stated that they had experienced IPV. As for the types of violence, 290 participants (52.7%) identified experiencing psychological violence, 119 (21.6%) physical violence, 114 (20.7%) moral violence, 74 (13.5%) patrimonial violence, and 63 (11.5%) sexual violence.

Concerning mental health outcomes, data showed that women with a history of IPV had a statistically significantly higher score of depression than those without a history (*t*(470) = 2.66, *p* = 0.001), with a moderate effect size (Cohen’s *d* = 0.44). Regarding stress indicators, we found that women with a history of IPV had higher scores (*M* = 17.44; *SD* = 5.28) than women without a history of IPV, with this difference being statistically significant (*t*(470) = 1.83, *p* = 0.001), and with a small effect size (Cohen’s *d* = 0.35). Finally, women with a history of IPV had higher levels of anxiety than women without a history, this difference being statistically significant (*t*(470) = 2.02, *p* = 0.001), with a small effect size (Cohen’s d = 0.36) (Table 1).

**Table 1:** Mental health indicators in women with and without a history of IPV

Indicator	M	SD	t	Gl	*P*-value	CI of Mean Difference (95%)
						Superior Inferior Limit Limit
Depression						
Suffered from IPV	16.832	6.407	2.664	470	0.001	1.626–3.715
Did not suffer from IPV	14.167	5.597				
Anxiety						
Suffered from IPV	14.850	5.999	2.017	470	0.001	0.966–3.045
Did not suffer from IPV	12.832	5.095				
Stress						
Suffered from IPV	17.439	5.279	1.831	470	0.001	0.935 - 2.812
Did not suffer from IPV	15.607	5.139				

*N* = 280 (suffered from IPV) *N* = 191 (did not suffer from IPV)

In terms of perceived social support, lesbian women with a history of IPV reported lower average support levels compared to those without a history. This difference was statistically significant (*t*(445) = 1.50, *p* = 0.002), with a small effect size (Cohen’s *d* = 0.20). Table 2 summarizes the findings on the relationship between social support perception and IPV. We also explored actions related to seeking support. Among 185 participants asked about formally reporting IPV to the police, only one participant (0.5%) pursued this option. However, this individual was not referred to the assistance network for further support. Regarding disclosure of IPV experiences, 139 participants (25.3%) shared their experiences with friends, 38 (6.9%) with family members, and 27 (4.9%) did not disclose to anyone.

**Table 2:** Social support in women with and without a history of IPV

Type of Support	M	SD	t	Gl	*P*-value	CI of Mean Difference (95%)
						Superior Inferior Limit Limit
Total social support						
Suffered from IPV	19.287	5.594	−1.504	445	0.002	−2.375 – −0.648
Did not suffer from IPV	20.792	4.657				
Support from friends						
Suffered from IPV	20.918	6.703	−1.686	445	0.006	−2.843 – −0.509
Did not suffer from IPV	22.605	5.839				
Support from family						
Suffered from IPV	15.483	7.500	−0.785	445	0.276	−2.135 – 0.647
Did not suffer from IPV	16.268	4.458				
Support from others						
Suffered from IPV	21.461	6.620	−2.041	445	0.001	−3.173 – −0.870
Did not suffer from IPV	23.502	5.635				

*N* = 280 (suffered from IPV) *N* = 191 (did not suffer from IPV)

The analysis of minority stress elements revealed distinct patterns among lesbian women with and without a history of IPV. Women with a history of IPV had lower mean scores of internalized homonegativity compared to those without; however, this difference was not statistically significant (*t*(348) = −2.88, *p* = 0.629). In contrast, women with a history of IPV reported significantly higher levels of stigma experiences imposed by others compared to women without a history of IPV (*t*(348) = 1.24, *p* = 0.001), with a moderate effect size (Cohen’s *d* = 0.45). Finally, regarding the concealment of sexuality, women with a history of IPV had significantly higher rates of concealment than those without, with this difference also being statistically significant (*t*(348) = 0.792, *p* = 0.01), and a small effect size (Cohen’s *d* = 0.32) (Table 3).

**Table 3:** Minority stress in women with and without a history of IPV

Stressor	M	SD	t	Gl	*P*-value	CI of Mean Difference (95%)
						Superior Inferior Limit Limit
Homonegativity Internalized						
Suffered from IPV	12.958	5.910	−0.288	348	0.628	−1.377 – 0.959
Did not suffer from IPV	13.247	5.527				
Stigma						
Suffered from IPV	10.569	3.177	1.245	348	0.001	0.740–1.728
Did not suffer from IPV	9.323	2.108				
Concealment of Sexuality						
Suffered from IPV	17.292	2.536	0.792	348	0.001	0.314–1.264
Did not suffer from IPV	16.500	2.471				

*N* = 280 (suffered from IPV) *N* = 191 (did not suffer from IPV)

Table 4. presents the results of the logistic regression model. Correlation analyses were conducted among sociodemographic variables, minority stress model variables, substance use, perception of social support, mental healthcare, and IPV. Variables that showed significant correlations were included in the model. In summary, the model significantly contributed to explaining intimate partner violence (IPV) among lesbian women, with *χ²*(4) = 54.19, *p* < 0.000, and Nagelkerke’s *R²* = 0.16. As presented in Table 4., age, experiences of stigma throughout life, perception of social support, and not being under mental healthcare emerged as significant predictors of IPV.

**Table 4.** IPV logistic regression model

Variable	*B*	*Standard Error*	*p*	*OR*	*OR CI 95%*
Constant	-0.169	0.753	0.805		
Age 18-24 years	0.897	0.213	0.001	2.453	0.450-1.385
Stigma	0.152	0.043	0.001	1.164	0.074-0.252
Perception of Social Support	-0.66	0.021	0.001	0.936	0.104-0.031
Mental Healthcare	-0.610	0.211	0.004	0.543	0.352-0.820

*OR* Odds Ratio. *ORCI* 95% = 95% confidence intervals of odds ratios

## Discussion

The first result to be discussed concerns the typologies and prevalence of IPV among the participants. Descriptive analyses revealed that the most frequent type of violence in the sample was psychological, followed by physical, moral, patrimonial, and sexual violence. This finding aligns with previous studies highlighting the predominance of psychological violence, followed by physical violence, in IPV cases among women (Longobardi & Ribera, 2017; Osorio et al., 2020; Whitfield et al., 2021). Other studies also report the presence of sexual and patrimonial violence (Longobardi & Ribera, 2017; Osorio et al., 2020; Whitfield et al., 2021). However, there is no Brazilian data to provide a basis for comparison. Regarding psychological violence, women subjected to this type may misinterpret its manifestations - such as name-calling, emotional extortion, and criticism - as responses to events affecting their emotional state or that of their partner, such as unemployment, chemical dependency, or family mourning (Silva et al., 2007). This misinterpretation may lead to justifying the aggression and impairing its recognition. Additionally, since psychological violence does not leave physical marks, it can be harder to identify, potentially contributing to its perpetuation and escalation into physical violence (Silva et al., 2007). This could explain the discrepancy in IPV prevalence in this sample before and after examples of violence types were provided.

With regard to mental health indexes, the results suggest that lesbian women who have a history of IPV have higher rates of mental health impairment – depression, anxiety, and stress – than those without a history, confirming our hypothesis. This possibly occurs due to the combination of social stressors related to gender and sexuality, which adds to the impacts of IPV. The data obtained are in line with the literature, as women with IPV history are more prone to mental illness (WHO, 2021). Furthermore, the results corroborate specific studies on the effects of IPV on the mental health of lesbian women (Gehring & Vaske, 2017; Miller & Irvin, 2016).

As for the perception of social support, the results showed that lesbian women with a history of IPV report lower levels of perceived support compared to those without such a history. These data confirm the hypothesis that Brazilian women with a history of IPV report receiving limited social support, which can also explain the worse outcomes in mental health. Lesbian women with low levels of perceived support are more vulnerable to IPV and, consequently, less likely to seek help (Furukawa et al., 2022). Social support is known to aid in coping with IPV and serves as a protective factor for mental health (Miller & Irvin, 2016; Zamora et al., 2021). However, a dysfunctional support network may lead to increased social isolation and hinder the disclosure of IPV experiences. The low perception of support can contribute to the phenomenon known as a “double closet” (Santos, 2012; Topa, 2010) and influence the process of seeking help from close people or the assistance system (Furukawa et al., 2022; Santoniccolo et al., 2023).

Having an adequate protection system can facilitate the process of disclosing IPV situations. In the sample, the main categories of support sources were “others” and “friends,” with friends being the primary source for disclosing IPV situations. Conversely, the “family members” category did not hold significant value as a support source and was less frequently sought for IPV disclosure compared to friends. This finding may be explained by experiences of discrimination within the family environment, prompting individuals to seek support from others, thereby highlighting the concept of a family of choice (Shilo et al., 2015).

When investigating the search for formal support for coping with IPV, the results showed that only one participant reported having sought help from the protection system – police station – and, still, she was not referred to a specialized assistance system. Based on this data, it becomes evident that the Brazilian protection system may not be prepared to help non-heterosexual women experiencing IPV and does not transmit security so that lesbian – and bisexual – women can seek proper support. In addition, we can infer that Brazilian lesbian women may be unaware of this type of place as a source of support, since campaigns to end violence against women are mostly formatted for women who have relationships with men (Santos et al., 2012; Santos et al., 2019). The fragmentation of the network’s actions can contribute to the re-victimization of women in situations of violence (Dutra et al., 2013) and, in cases of IPV amongst lesbian women, there may be an even greater distance of the reality of the demands, contributing as a barrier for them to access their rights (Luz & Gonçalves, 2015).

These barriers in the pursuit of rights are closely linked to minority stress related to sexual orientation and gender. Based on the results, lesbian women who have experienced IPV reported higher levels of stigma and concealment of sexual orientation compared to those without such a history. This underscores how lesbian women are subjected to community violence rooted in gender and sexuality based oppression, reinforcing the understanding of IPV as a social issue (Santos, 2012; Topa, 2010). Beyond IPV-related stressors, they also face prejudice and the lack of legitimacy and recognition of their relationships, which pose significant barriers to accessing rights and support (Santos, 2012; Santos, 2019; Souza et al., 2021a, b). These barriers contribute to the “double closet” phenomenon, where reporting abuse also requires disclosing one’s sexual orientation, intensifying stress and psychological distress. Within this dynamic, psychological violence may involve threats by the partner to out the victim as a form of control (DiStefano, 2009; Topa, 2010). This may help explain the higher levels of sexual orientation concealment and the poorer mental health outcomes observed among participants with a history of IPV in our sample. The double closet aligns with Meyer’s (2003) concept of proximal stressors, particularly concealment, which has a significant impact on mental health.

Notwithstanding other predictors, age also played a significant role: younger women (18 to 24 years old) were more likely to experience IPV. Although this outcome has been previously identified in studies conducted in the Global North (DiStefano, 2009; WHO, 2021), this is among the first studies to report similar findings in a Brazilian Latin American sample of lesbian women. Significant power dynamics may emerge in relationships with age differences, as age can be associated with positions of dominance or perceived superiority between partners. (DiStefano, 2009). In relationships between women, the exertion of power over a partner manifests differently compared to relationships between a man and a woman. Additionally, other social markers, such as gender identity, race, and others, can function as factors of domination due to potential power imbalances within these relationships (Harden et al., 2022).

Other predictors of IPV included a low perception of social support and experiences of social stigma. As discussed previously, an adequate support network can facilitate coping with IPV (Zamora et al., 2021), and a sense of belonging to the community contributes to greater well-being among LGBTQ + individuals (Meyer, 2003; Shilo et al., 2015). Conversely, a low perception of social support can have the opposite effect, increasing vulnerability to IPV and its perpetuation (Furukawa et al., 2022). Among the elements of minority stress, only the experience of stigma significantly predicted IPV. Lesbian women are more vulnerable to violence due to intersecting gender and sexuality-based prejudice (Santos, 2012; Souza et al., 2021a, b), which may heighten emotional distress and contribute to IPV. One possible reason internalized homonegativity and concealment of sexual orientation were not predictors of IPV is the protective effect of community belonging, which can buffer stigma’s impact and promote positive self-perception (Meyer, 2003). This is conesistent with the findings, as “others” and “friends” were the most significant sources of perceived social support in the sample. Lesbian women may experience stronger ties within their own community, reinforcing a sense of belonging and resilience (Paveltchuk & Borsa, 2019). Additionally, the dissemination of this research through platforms targeting lesbian and LGBTQ + communities may have contributed to a stronger sense of community among participants, reducing levels of self-rejection and internalized negativity.

Although the literature identifies race (Harden et al., 2022; Whitfield et al., 2021) and social class (Harden et al., 2022) as predictors of IPV in relationships among women, and IPV rates in the Brazilian population indicate a higher prevalence of victimization among Black women (Secretaria de Vigilância em Saúde, 2020), the statistical analysis did not show significant values for these markers as IPV predictors in the sample. This may be attributed to the socioeconomic composition of the sample, as most participants reported favorable financial conditions, with fewer women belonging to lower-income groups. Furthermore, the predominance of Brazilian white women in the sample may have introduced a bias, limiting the generalizability of the findings. This highlights the need for further studies with more diverse samples to fully explore how race and social class influence IPV dynamics among Brazilian lesbian women. Future research should aim to address these variables comprehensively and investigate their potential intersections with IPV.

### Limitations

As limitations of this study, we highlight the transversal design and suggest that longitudinal studies can be carried out within the Brazilian context in order to better understand IPV amongst lesbian women. We also highlight a possible sample bias, because most of the participants were Brazilian white women, residents of urban areas, and that were not part of a low socioeconomic status. Furthermore, the instrument used to assess the impacts of MS comprises the same expressions for men and women, disregarding particularities of gender and sexuality. Because of this, some of the expressions can generate detachment from the reality experienced by the target audience, since lesbian and bisexual women and gay and bisexual men have different stressors regarding gender and sexuality.

Moreover, all participants were cisgender women and, therefore, it is essential that future studies include transgender women who identify as lesbians, and that more research be conducted to understand how IPV affects their experiences. . Finally, the instrument used to assess social support has the category *others* but does not offer the possibility for the participants to describe which people constitute it – it presumably contemplates support network professionals, the current partner, community members, and other significant people. It could be beneficial for new studies to understand in more depth what comprises this category and also explore other more robust quantitative methodologies, such as longitudinal studies, as well as qualitative strategies.

## Conclusion

Understanding the dynamics of IPV among Brazilian lesbian women is essential for providing effective care and addressing this complex health issue. This study found that psychological violence was the most prevalent form of abuse, followed by physical violence. Psychological violence in lesbian relationships can manifest in various ways, such as through extortion and threats to expose a partner’s sexual orientation (DiStefano, 2009; Topa, 2010). Recognizing these dynamics is crucial for guiding effective psychological care that does not contribute to the invisibility or neglect of Brazilian lesbian women’s needs. The findings of this study highlight how IPV can further jeopardize the mental health of Brazilian lesbian women, exacerbating symptoms of stress, depression, and anxiety. Discriminatory and stigmatizing acts amplify the effects of IPV, as violence is perpetrated on both social and individual levels.

Due to the recurrent social stigma, Brazilian lesbian women feel discouraged from seeking help from the formal network and support systems, because is not unusual that the professionals, including psychotherapists, engage in discriminatory behavior directly (e.g.: denying care) or indirectly (e.g.: assuming that every woman seeking support is heterosexual, for instance). Therefore, professional qualification is necessary for affirmative practices to be effective, for increasing the demand for support, and for the provision of adequate care. Also, it is essential to decentralize campaigns to end violence against women that are aimed at heterosexual women, so that other audiences can be served, facilitating greater adherence to support and knowledge about the subject.

Lesbian women are often not recognized in their sexuality, and their relationships are frequently considered illegitimate by society. This is also the case within academia, where scientific research often overlooks their existence, as well as that of other non-heterosexual or non-cisgender individuals. There is a lack of Brazilian studies on IPV in intimate relationships among women, which becomes even more limited when specifically addressing lesbian women. We advocate for more research on IPV using Brazilian samples to make this violence visible and fight against it. Additionally, innovative studies are needed to develop public policies that specifically address the particularities of IPV among women. In a country where social violence is legitimized, especially in recent years, actions to combat and confront this issue are essential. Conducting research on this topic can provide a deeper understanding of IPV among lesbian women and support evidence-based interventions aimed at addressing and preventing this phenomenon. Thus, producing knowledge is also a way of actively combating the violence and prejudice faced by lesbian women.

## Data Availability

the datasets generated and/or analyzed in the current study are not publicly available due to ethical procedures, since participants signed a data sharing agreement solely for research purposes and have not agreed for the data to be shared with third parties. If necessary, the dataset can be made available from the corresponding author upon reasonable request.

## Abbreviations

IPV: The intimate partner violence
LGBTQ+: Lesbian, gay, bisexual, transgender, queer persons
LGB: lesbian, gay, and bisexual
MST: Minority Stress Theory
MS: Minority Stress

## Declarations

We confirm that the content of the manuscript has not been published or submitted for publication elsewhere and that there are no issues relating to journal policies.
